# CD300LF Polymorphisms of Inbred Mouse Strains Confer Resistance to Murine Norovirus Infection in a Cell Type-Dependent Manner

**DOI:** 10.1128/JVI.00837-20

**Published:** 2020-08-17

**Authors:** Kevin Furlong, Scott B. Biering, Jayoung Choi, Craig B. Wilen, Robert C. Orchard, Christiane E. Wobus, Christopher A. Nelson, Daved H. Fremont, Megan T. Baldridge, Glenn Randall, Seungmin Hwang

**Affiliations:** a Committee on Microbiology, The University of Chicago, Chicago, Illinois, USA; b Department of Pathology, The University of Chicago, Chicago, Illinois, USA; c Department of Laboratory Medicine, Yale University School of Medicine, New Haven, Connecticut, USA; d Department of Immunobiology, Yale University School of Medicine, New Haven, Connecticut, USA; e Department of Immunology, The University of Texas Southwestern Medical Center, Dallas, Texas, USA; f Department of Microbiology and Immunology, University of Michigan, Ann Arbor, Michigan, USA; g Department of Pathology & Immunology, Washington University, St. Louis, Missouri, USA; h Department of Biochemistry & Molecular Biophysics, Washington University, St. Louis, Missouri, USA; i Department of Molecular Microbiology, Washington University, St. Louis, Missouri, USA; j Division of Infectious Diseases, Department of Medicine, Washington University School of Medicine, St. Louis, Missouri, USA; k Department of Microbiology, The University of Chicago, Chicago, Illinois, USA; Hudson Institute of Medical Research

**Keywords:** CD300LF, I/LnJ, entry, norovirus, polymorphism, receptors

## Abstract

Human norovirus is the leading cause of gastroenteritis worldwide, yet basic questions about its life cycle remain unanswered due to an historical lack of robust experimental systems. Recent studies on the closely related murine norovirus (MNV) have identified CD300LF as an indispensable entry factor for MNV. We compared the MNV susceptibilities of cells from different mouse strains and identified polymorphisms in murine CD300LF which are critical for its function as an MNV receptor. Bone marrow-derived macrophages (BMDMs) from I/LnJ mice were resistant to infection from multiple MNV strains which readily infect BMDMs from C57BL/6J mice. The resistance of I/LnJ BMDMs was specific to MNV, since the cells supported infection of other viruses comparably to C57BL/6J BMDMs. Transduction of I/LnJ BMDMs with C57BL/6J CD300LF made the cells permissible to MNV infection, suggesting that the cause of resistance lies in the entry step of MNV infection. In fact, we mapped this phenotype to a 4-amino-acid difference at the CC′ loop of CD300LF; swapping of these amino acids between C57BL/6J and I/LnJ CD300LF proteins made the mutant C57BL/6J CD300LF functionally impaired and the corresponding mutant of I/LnJ CD300LF functional as an MNV entry factor. Surprisingly, expression of the I/LnJ CD300LF in other cell types made the cells infectible by MNV, even though the I/LnJ allele did not function as an MNV receptor in macrophage-like cells. Correspondingly, I/LnJ CD300LF bound MNV virions in permissive cells but not in nonpermissive cells. Collectively, our data suggest the existence of a cell type-specific modifier of MNV entry.

**IMPORTANCE** MNV is a prevalent model system for studying human norovirus, which is the leading cause of gastroenteritis worldwide and thus a sizeable public health burden. Elucidating mechanisms underlying susceptibility of host cells to MNV infection can lead to insights on the roles that specific cell types play during norovirus pathogenesis. Here, we show that different alleles of the proteinaceous receptor for MNV, CD300LF, function in a cell type-dependent manner. In contrast to the C57BL/6J allele, which functions as an MNV entry factor in all tested cell types, including human cells, I/LnJ CD300LF does not function as an MNV entry factor in macrophage-like cells but does allow MNV entry in other cell types. Together, these observations indicate the existence of cell type-specific modifiers of CD300LF-dependent MNV entry.

## Abstract (precis)

MNV is a prevalent model system for studying human norovirus, which is the leading cause of gastroenteritis worldwide and thus a sizeable public health burden. Elucidating mechanisms underlying susceptibility of host cells to MNV infection can lead to insights on the roles that specific cell types play during norovirus pathogenesis. Here, we show that different alleles of the proteinaceous receptor for MNV, CD300LF, function in a cell type-dependent manner. In contrast to the C57BL/6J allele, which functions as an MNV entry factor in all tested cell types, including human cells, I/LnJ CD300LF does not function as an MNV entry factor in macrophage-like cells but does allow MNV entry in other cell types. Together, these observations indicate the existence of cell type-specific modifiers of CD300LF-dependent MNV entry.

## INTRODUCTION

Human norovirus (HNV) is a nonenveloped, positive-sense RNA virus of the *Caliciviridae* family and is the leading cause of acute gastroenteritis worldwide (1–3). Despite its significant public health burden, a complete understanding of the host factors controlling the life cycle of HNV is still lacking. Currently, there are few *in vitro* models that support replication and detection of HNV, making it a difficult pathogen to study directly, though these systems are rapidly improving (1, 4–7). Murine norovirus (MNV) is a genetically similar virus discovered in 2003 as a lethal agent in *Rag2*^−/−^ *Stat1*^−/−^ mice and has since been used as a model virus to study HNV biology (8). Unlike HNV, MNV replicates robustly in several macrophage-like cell lines, including BV2 and RAW264.7 (9, 10).

Several studies have identified a wide range of host factors that modulate norovirus attachment and entry, including histo-blood group antigens (HBGAs), bile acids, sialic acid, and divalent cations (9, 11–14). While these attachment factors have been shown to enhance attachment for several different noroviruses, none of them are required for MNV infection. In contrast, recent clustered regularly interspaced short palindromic repeat (CRISPR)/Cas9 screens uncovered CD300LF, a type I integral membrane protein containing a single immunoglobulin-like domain, as an indispensable host factor for MNV attachment and entry (9, 10, 15). In fact, expression of murine CD300LF alone was sufficient to confer MNV susceptibility to otherwise resistant host cells, including those from other species, such as human 293T and HeLa cells (9, 10). Additional receptor molecules and attachment factors have been identified for closely related members of the *Caliciviridae* family, including feline junctional adhesion molecule A (fJAM-A) as the receptor for feline calicivirus, which has been used historically as a surrogate for HNV (16–23). As understanding the mechanisms by which viruses enter susceptible host cells is integral to understanding the viral life cycle, recent studies on MNV entry have significantly advanced our understanding of norovirus biology (9, 10, 15, 24–26). Nevertheless, the modulation of norovirus entry factors and their mode of interaction with the viruses are still unclear, and it remains to be determined how these factors underlie norovirus host cell tropism.

The study of how genetically divergent hosts respond to viral infections can reveal the importance of host genetic factors, which may not be evident when using a single strain (27). With many cellular factors influencing norovirus infection, we asked if hosts from different genetic backgrounds might have different susceptibilities to MNV. Variation exists in the protein sequences of different mouse strains, and these polymorphisms can help elucidate the functions of certain proteins. Here, we show that bone marrow-derived macrophages (BMDMs) from two different mouse strains have dramatically different susceptibilities to MNV infection. We found that these different susceptibilities are primarily due to divergence in the CC′ loop domain of CD300LF, which is essential for its function as an MNV receptor (9). Surprisingly, the CD300LF variant that cannot function as an MNV receptor in macrophage-like cells is able to bind MNV virions and is functional as an MNV receptor in different cell types. These data suggest the existence of cell type-specific modifiers of CD300LF-MNV interactions during viral entry.

## RESULTS

### I/LnJ BMDMs resist MNV infection.

**FIG 1:**
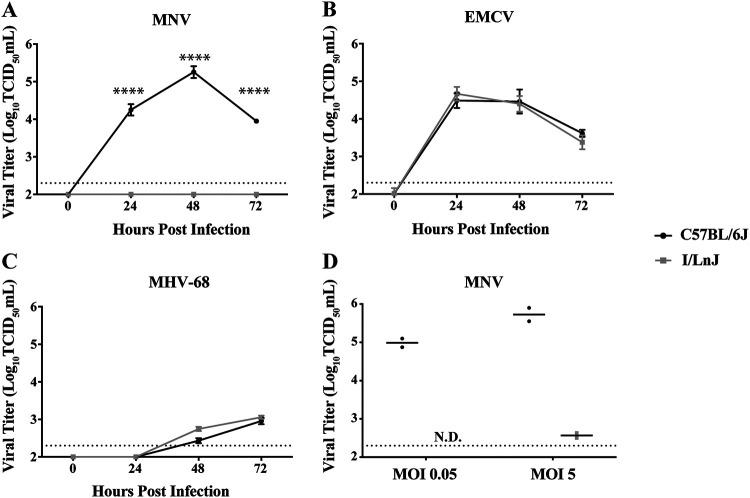
I/LnJ BMDMs resist MNV infection. (A to C) Analysis of replication kinetics of MNV (A), EMCV (B), and MHV-68 (C) in C57BL/6J and I/LnJ BMDMs. Cells were inoculated at a multiplicity of infection (MOI) of 0.05 TCID_50_/cell and harvested at the indicated time points to determine the titers infectious viruses via TCID_50_ assay in BV2 cells. All experiments were done in triplicates, and data are presented as mean ± standard error of the mean (SEM). (D) Analysis of MNV replication at 24 h postinfection in C57BL/6J and I/LnJ BMDMs after inoculation at MOIs of 0.05 and 5 TCID50/cell. The experiment was done twice, and data are presented as dots with mean as a bar. N.D., not detected.

Inbred mouse strains show differences in innate susceptibility to viral infections (28). While examining the susceptibility of BMDMs from different mouse strains to MNV inoculation, we found that BMDMs derived from I/LnJ mice, which are resistant to mouse retroviruses (29), were completely resistant to MNV infection. Such resistance is in strong contrast to the case for C57BL/6J BMDMs, which support robust MNV infection (Fig. 1A). To investigate the specificity of the resistance of I/LnJ BMDMs to MNV infection, we examined the replication of encephalomyocarditis virus (EMCV), as another virus with a positive-sense RNA genome, and of murine gammaherpesvirus 68 (MHV-68), as a virus with a DNA genome. In contrast to that of MNV, the replication of both EMCV and MHV-68 was supported in C57BL/6J and I/LnJ BMDMs with similar growth kinetics (Fig. 1B and C). We also performed infection with a high multiplicity of infection (MOI) (5) and did not detect any sign of MNV replication in I/LnJ BMDMs other than remaining input virus (Fig. 1D). These results suggest that the inability of I/LnJ BMDMs to support viral replication is specific to MNV.

**FIG 2:**
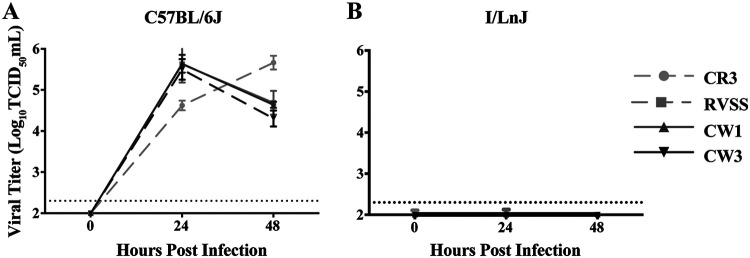
I/LnJ BMDMs are resistant to different strains of MNV. Analysis of replication kinetics of MNV strains CR3, RVSS, CW1, and CW3 in C57BL/6J (A) or I/LnJ (B) BMDMs is shown. Cells were inoculated at an MOI of 0.05 TCID_50_/cell and harvested at the indicated time points to determine the titer of infectious viruses via TCID_50_ assay in BV2 cells. All experiments were done in triplicates, and data are presented as mean ± SEM.

Different strains of MNV are known to bind glycoproteins differentially, which can lead to different levels of infection *in vivo* (30). To examine whether I/LnJ BMDMs are resistant to a broad range of MNV strains, we inoculated C57BL/6J and I/LnJ BMDMs with several strains of MNV and measured viral replication over 48 h. Specifically, in addition to the CW3 strain, we chose CW1, RVSS, and CR3. CW1 is a plaque isolate of MNV-1, RVSS is a mutant strain of CW1 with an altered glycoprotein binding profile, and CR3 is a field isolate with over 95% sequence identity with the capsid regions of CW1 and CW3 (30, 31). Similar to the result with strain CW3, I/LnJ BMDMs were resistant to infection with all MNV strains tested (Fig. 2A and B). These data demonstrated that I/LnJ BMDMs are resistant to multiple MNV isolates, suggesting a general resistance of I/LnJ BMDMs to MNV infection.

### I/LnJ BMDMs expressing C57BL/6J CD300LF are susceptible to MNV infection.

**FIG 3:**
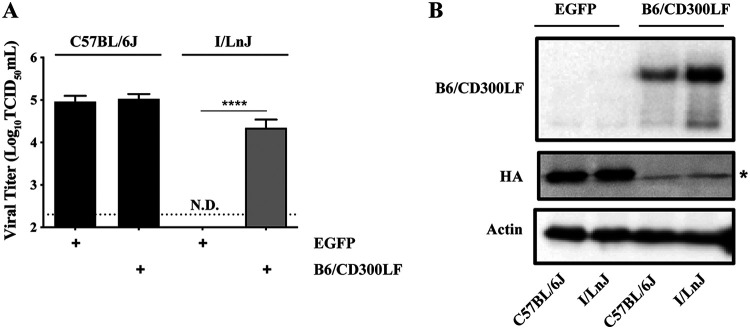
I/LnJ BMDMs expressing C57BL/6J CD300LF are susceptible to MNV infection. (A) Comparison of MNV replication in C57BL/6J and I/LnJ BMDMs transduced with lentivirus expressing EGFP (control) or the C57BL/6J allele of CD300LF. Transduced cells were inoculated with MNV at an MOI of 0.05 TCID_50_/cell and harvested at 24 h postinfection to determine the titer of infectious viruses via TCID_50_ assay in BV2 cells. All experiments were done in triplicates, and data are presented as mean ± SEM. N.D., not detected. (B) A representative Western blot of C57BL/6J and I/LnJ BMDMs transduced with lentivirus expressing EGFP (control) or the C57BL/6J allele of CD300LF as detected with goat anti-mouse CD300LF (R&D systems, AF2788). *, nonspecific signal in the blot. Actin is included as a loading control; *n* = 3 replicates.

Recently, unbiased CRISPR/Cas9 screenings of host factors for MNV infection identified CD300LF as a host factor to mediate MNV entry into cells (9, 10). Given the requirement of CD300LF for MNV entry coupled with the complete resistance of I/LnJ BMDMs to MNV infection, we speculated that I/LnJ BMDMs might fail to express a functional CD300LF receptor required for MNV to infect cells. Therefore, we transduced BMDMs from C57BL/6J and I/LnJ mice with lentiviruses expressing either the C57BL/6J allele of CD300LF (B6/CD300LF) that was shown to mediate MNV entry (9) or enhanced green fluorescent protein (EGFP) as a control. Expression of B6/CD300LF in C57BL/6J BMDMs did not significantly affect susceptibility of the cells to MNV infection. In contrast, I/LnJ BMDMs became susceptible to MNV infection upon expression of B6/CD300LF (Fig. 3A and B). These data clearly demonstrated that the resistance of I/LnJ BMDMs to MNV infection was largely, if not completely, at the step of viral entry, suggesting a defect of I/LnJ BMDMs in expressing functional MNV receptor CD300LF.

### A cluster of four consecutive amino acids determines the functionality of CD300LF to support MNV infection.

The difference in susceptibility to MNV infection between I/LnJ and C57BL/6J BMDMs could reflect a simple difference in the cell surface expression of CD300LF or genetic variations within the protein itself. Unfortunately, none of the currently available antibodies for CD300LF could detect I/LnJ CD300LF, and we have been unable to detect surface expression of endogenous CD300LF in I/LnJ BMDMs. Thus, we examined publicly available sequences of murine CD300LF proteins to determine how the different alleles of CD300LF vary from each other and whether these differences could contribute to the functionality of the proteins. When we compared the sequences of CD300LF proteins from the C57BL/6J and I/LnJ mouse strains, we found that the C57BL/6J and I/LnJ variants of CD300LF differ by 14 amino acids, 9 of them in the regions important for function as a receptor for MNV (9). We hypothesized that the sequence difference in CD300LF proteins could underlie the inability of I/LnJ CD300LF to function as an MNV receptor in BMDMs.

**FIG 4:**
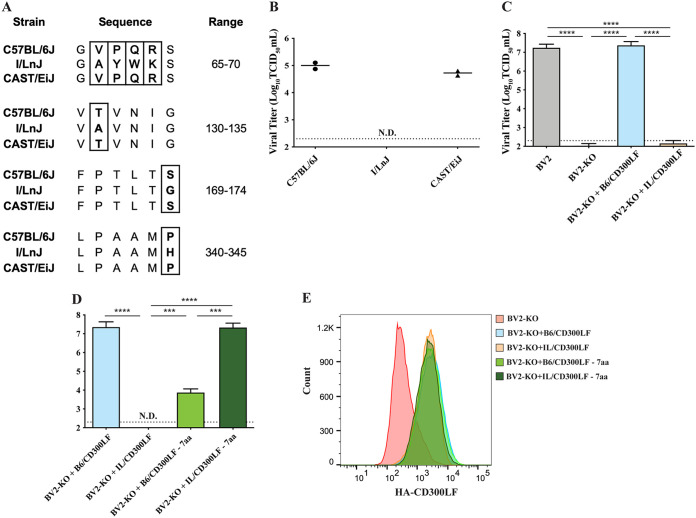
Identification of seven amino acids determining the functionality of CD300LF as an MNV receptor. (A) Amino acid sequences for the CD300LF proteins of the indicated mouse strains at the indicated ranges. Boxed sequences denote the amino acids of CAST/EiJ CD300LF that are different from those of I/LnJ CD300LF but identical to those of C57BL6J CD300LF. (B) Comparison of MNV replication in C57BL/6J, I/LnJ, or Cast/EiJ BMDMS. Cells were inoculated with MNV at an MOI of 0.05 TCID_50_/cell and harvested at 24 h postinfection to determine the titer of infectious viruses via TCID_50_ assay in BV2 cells. The experiment was done twice, and data are presented as dots with mean as a bar. N.D., not detected. (C) Comparison of MNV replication in BV2 cells. BV2 cells, BV2 cells with endogenous *Cd300lf* knocked out (BV2-KO), or BV2-KO cells transduced with lentiviruses expressing either C57BL/6J or I/LnJ CD300LF were inoculated with MNV at an MOI of 5 TCID_50_/cell and harvested at 24 h postinfection to determine the titer of infectious viruses via TCID_50_ assay in BV2 cells. All experiments were done in triplicates, and data are presented as mean ± SEM. (D) Comparison of MNV replication in BV2-KO cells transduced with WT or 7aa swapped constructs. Cells were inoculated with MNV at an MOI of 5 TCID_50_/cell and harvested at 24 h postinfection to determine the titer of infectious viruses via TCID_50_ assay in BV2 cells. All experiments were done in triplicates, and data are presented as mean ± SEM. N.D., not detected. (E) Analysis of surface expression of transduced CD300LF of the indicated background in BV2-KO cells. A representative image from three independent experiments is shown.

To determine the amino acids that could potentially be responsible for this MNV-resistant phenotype of I/LnJ CD300LF, we sought a mouse strain that could serve as a “genetic intermediate” between C57BL/6J and I/LnJ. The CD300LF sequences of the CAST/EiJ and I/LnJ mouse strains differ by 11 amino acids; 7 of these 11 sites are identical between the CAST/EiJ and C57BL/6J CD300LF proteins, but the other 4 amino acids are not identical between the two strains (Fig. 4A). To test whether the CD300LF allele of CAST/EiJ mice could serve as a receptor for MNV, we assessed whether BMDMs from the CAST/EiJ mouse are susceptible to MNV infection. In contrast to those from the I/LnJ strain, BMDMs from the CAST/EiJ strain supported MNV replication (Fig. 4B). These data suggest that the seven amino acids that are identical in CAST/EiJ and C57BL/6J CD300LF but different in I/LnJ CD300LF might be responsible for the MNV-resistant phenotype of I/LnJ BMDMs.

To expedite the mapping process, we also examined whether the highly tractable macrophage-like BV2 cell line could mimic the phenotypic difference between C57BL/6J and I/LnJ BMDMs (9). We transduced *Cd300lf*^−/−^ BV2 (BV2-KO) cells with lentiviruses expressing the hemagglutinin (HA)-tagged CD300LF protein of either C57BL/6J or I/LnJ. While C57BL/6J CD300LF (B6/CD300LF) expression confers MNV infection capacity to BV2-KO cells, expression of I/LnJ CD300LF (IL/CD300LF) is insufficient to permit infection of BV-2KO cells (Fig. 4C), similar to the results obtained using BMDMs (Fig. 1 and 2). To determine the role of the CAST/EiJ-identified seven amino acids in MNV infection, we expressed chimeric alleles of CD300LF: an otherwise wild-type (WT) C57BL/6J CD300LF with the seven amino acids of I/LnJ CD300LF at the CAST/EiJ-identified loci (B6/CD300LF-7aa) and the converse (IL/CD300LF-7aa). BV2-KO cells expressing B6/CD300LF-7aa had a significant reduction in their ability to support MNV replication (Fig. 4D). In contrast, BV2-KO cells expressing IL/CD300LF-7aa supported MNV replication like the BV2-KO cells with B6/CD300LF. All CD300LF proteins were expressed similarly on the cell surface (Fig. 4E). Collectively, these data suggested that the seven-amino-acid difference of the I/LnJ CD300LF from that of CD57BL/6J is responsible for the majority of the resistance of I/LnJ BMDMs to MNV infection.

**FIG 5:**
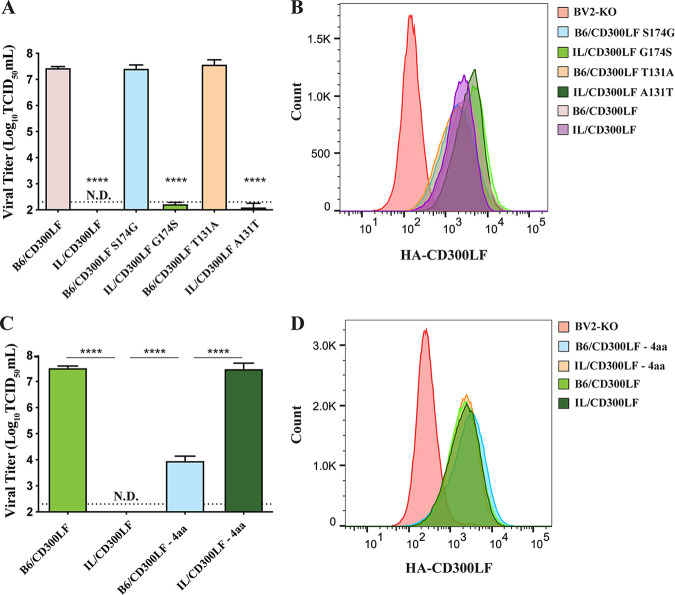
A cluster of four amino acids determines the functionality of CD300LF as an MNV receptor. (A and C) Analysis of MNV replication in BV2-KO cells transduced with the indicated mutant alleles of CD300LF. Cells were inoculated with MNV at an MOI of 5 TCID_50_/cell and harvested at 24 h postinfection to determine the titer of infectious viruses via TCID_50_ assay in BV2 cells. All experiments were done in triplicates, and data are presented as mean ± SEM. N.D., not detected. (B and D) Analysis of surface expression of HA-tagged CD300LF mutants in the indicated cells. Data shown are representative plots from three independent experiments. Asterisks indicate significance compared to B6/CD300LF. ns, not significant.

**FIG 6:**
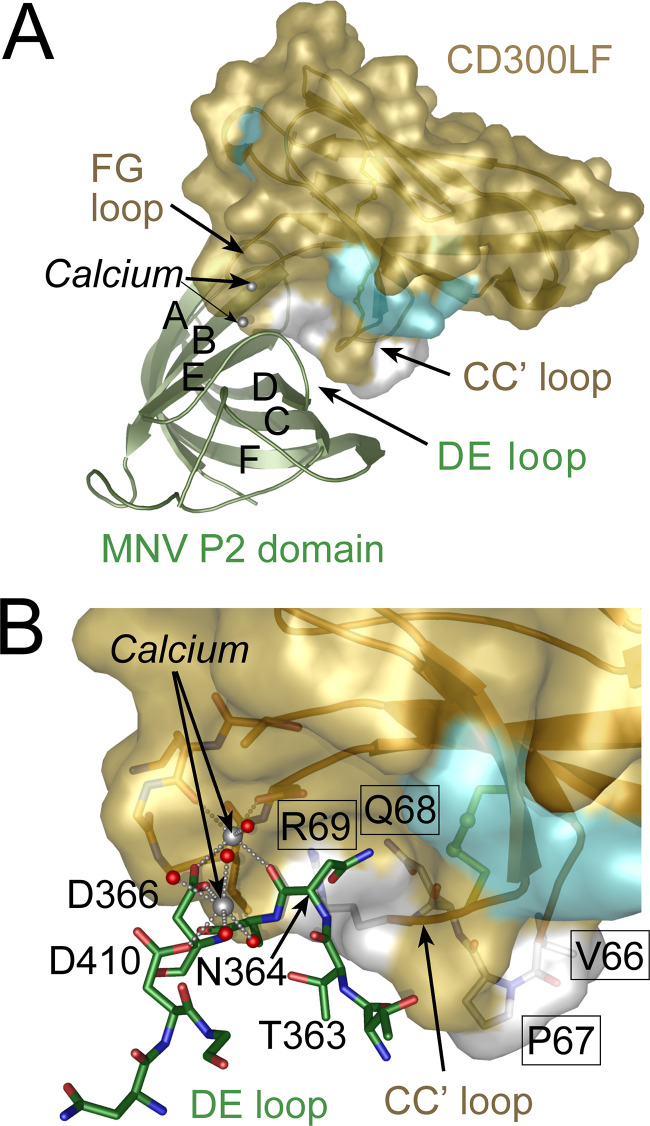
Residues critical for MNV infection occur in the CC′ loop flanking the phospholipid binding pocket of CD300LF. (A) Model showing the orientation of the MNV major capsid protein (VP1) P2 subdomain relative to CD300LF in complex with bound calcium ion. Positions that differ in sequence between B6/CD300LF and IL/CD300LF are colored white or cyan on the B6/CD300LF surface. (B) Close-up of the CD300LF-P domain complex showing the side chain of Asn364 from the P domain DE loop binding to CD300LF in a pocket made by the CC′ loop. The carbonyl oxygen of Asn364 coordinates with a calcium ion at the binding interface. A second calcium ion supports the P domain DE loop, holding it against the binding pocket, by bridging Asp410 and Asp366. The four residues in the CC' loop that differ between B6/CD300LF and IL/CD300LF are labeled with residue numbers in black boxes, and their side chain carbon atoms are colored in white. The area contributed by these residues is in white on the solvent-accessible surface. Most of the hydrophobic pocket shown to bind PC choline is contributed by the CD300LF residues Q68 and R69. Binding of the viral P2 domain to CD300LF is metal ion dependent and requires the DE loop of P2 to associate with the FG and CC' loops of CD300LF through a network of metal ion coordination and hydrogen bonding centered on Asn364. Substitution of the four critical residues in the CC' loop (VPQR to AYWK) almost certainly perturbs conformation of the CC' loop, disrupting the receptor binding interface. The four consecutive amino acids in the CC' loop correspond to positions 39 to 42 in the B6/CD300LF crystal structure (PDB accession no. 6E48).

We next set out to determine which combination of these seven amino acids is responsible for the resistance phenotype; we chose to prioritize six of the seven, as they are in the extracellular domain of CD300LF. According to software predictive of O-glycosylation, NetOGlyc (DTU Bioinformatics), two of the seven amino acid variations in B6/CD300LF had a high probability to be O-glycosylated. Since differential glycosylation status has been linked to the susceptibility of hosts to norovirus infection (12, 32), we examined these loci first. Expression of CD300LF mutants swapped at these potential glycosylation sites did not switch the phenotype of B6/CD300LF and IL/CD300LF in BV2-KO (Fig. 5A); that is, B6/CD300LF mutants with the I/LnJ amino acid sequence at the potential glycosylation sites (B6/CD300LF S174G and T131A) still made BV2-KO cells infectible by MNV, and expression of the corresponding I/LnJ CD300LF mutants (IL/CD300LF G174S and A131T) did not, despite similar levels of cell surface expression (Fig. 5B). The remaining difference in the extracellular domain of the CD300LF alleles was four consecutive amino acids at the CC′ loop, which flanks a phospholipid binding pocket along with the CDR3 domain and is critical for MNV infection (9, 24). When we swapped these four amino acids between C57BL/6J and I/LnJ CD300LF, the respective mutants, B6/CD300LF-4aa and IL/CD300LF-4aa, recapitulated the phenotype of the seven-amino-acid-swapped mutants in BV2-KO cells (Fig. 5C; phenotypes summarized in Table 1). Again, a similar level of cell surface expression was observed for all constructs (Fig. 5D). Taken together, these data demonstrated that the four-amino-acid polymorphisms at the CC′ loop of I/LnJ CD300LF is primarily responsible for its nonfunctionality as an MNV receptor in macrophage-like cells (Fig. 6).

**TABLE 1:**
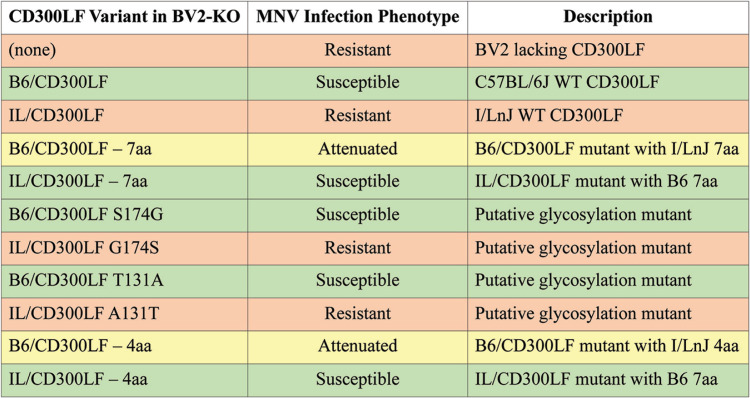
BV2 variant infection phenotypes^*a*^

a Resistant, no growth of MNV, as in I/LnJ BMDMs; attenuated, reduced growth of MNV compared to that in C57BL/6J BMDMs; susceptible: growth of MNV, as in C57BL/6J BMDMs.

### I/LnJ CD300LF can function as an MNV receptor in a cell type-dependent manner.

**FIG 7:**
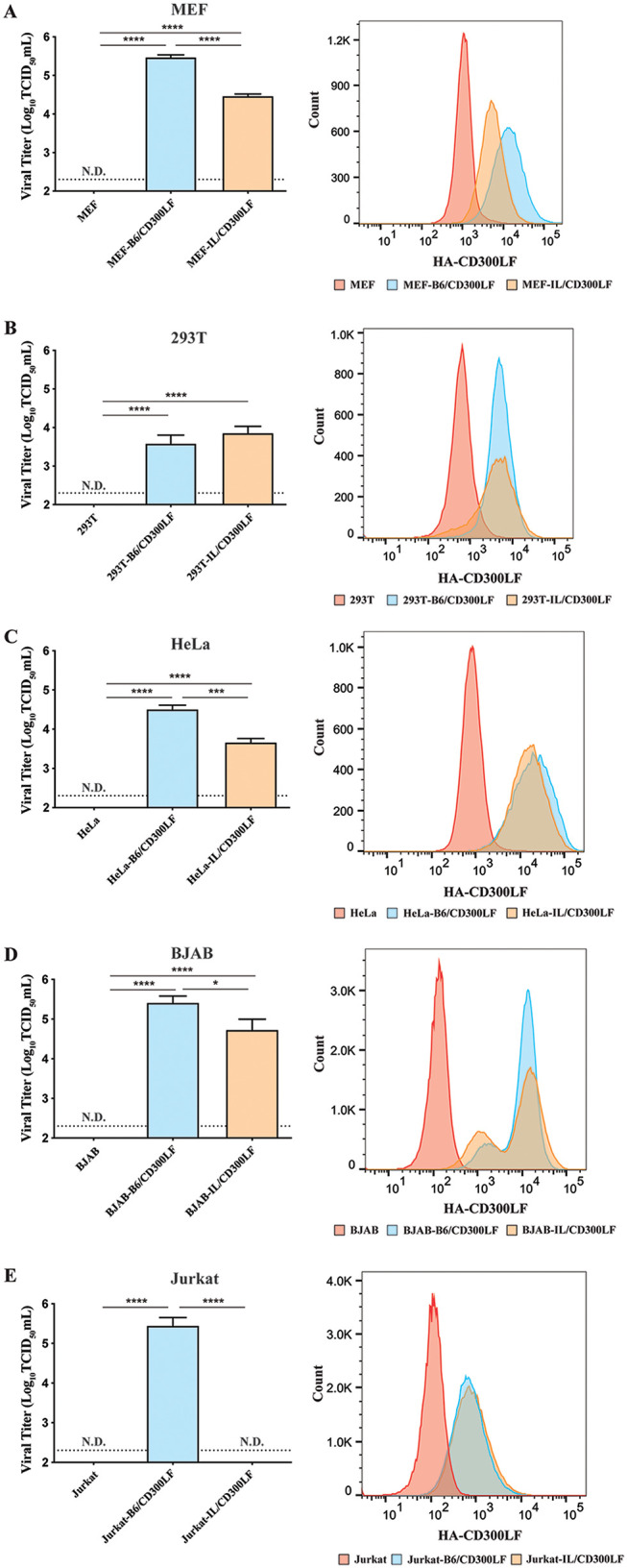
I/LnJ CD300LF can function as an MNV receptor in a cell type-dependent manner. (Left) Analysis of MNV replication in MEF (A), 293T (B), HeLa (C), BJAB (D), and Jurkat (E) cell lines transduced with the indicated allele of CD300LF. Cells were inoculated with MNV at an MOI of 5 (A, B, and C) or 0.1 (D and E) TCID_50_/cell and harvested at 24 h postinfection to determine the titer of infectious viruses via TCID_50_ assay in BV2 cells. Untransduced cells were included as a control. All experiments were done in triplicates, and data are presented as mean ± SEM. N.D., not detected. (Right) Analysis of surface expression of HA-tagged CD300LF alleles in the indicated cells. A representative plot from three independent experiments is shown.

Expression of C57BL/6J CD300LF in cells noninfectible by MNV makes the cells permissive to MNV infection, demonstrating the entry step as a major barrier for MNV infection (9). We examined whether I/LnJ CD300LF functions similarly in cell lines other than macrophage-like cells. Thus, we stably expressed C57BL/6J or I/LnJ CD300LF via lentiviral transduction in a variety of cell types that do not express murine CD300LF and thus are not susceptible to MNV infection, including mouse embryonic fibroblasts (MEFs) and human cell lines 293T, HeLa, BJAB, and Jurkat. CD300LF was expressed on the cell surface of all transduced cells (Fig. 7). Strikingly, while I/LnJ CD300LF did not function as an entry factor for MNV infection in BMDMs and BV2 cells, its expression in MEFs and in 293T, HeLa, and BJAB cells made the cells permissive for MNV replication (Fig. 7A to D). In human Jurkat T cells, however, only the expression of C57BL/6J CD300LF, and not that of I/LnJ CD300LF, made the cells susceptible to MNV infection, reminiscent of the macrophage-like phenotype (Fig. 7E). Taking the results together, while C57BL/6J CD300LF expression is necessary and sufficient for MNV infection in all contexts tested, the cell type-dependent functionality of I/LnJ CD300LF suggests the necessity of additional cell type-specific modifiers mediating MNV entry.

**FIG 8:**
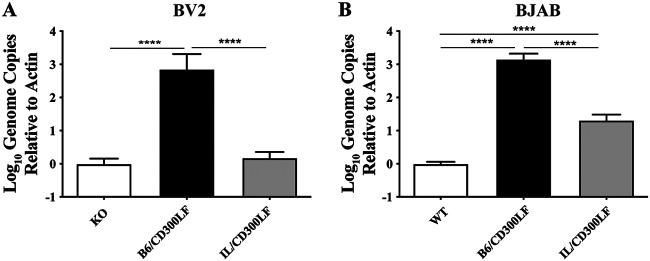
I/LnJ CD300LF supports MNV attachment in a cell type-dependent manner. Analysis of MNV binding to BV2 cells (A) or BJAB cells (B) expressing the indicated allele of CD300LF is shown. Cells were inoculated at 4°C at an MOI of 2 TCID_50_/cell. At 1 h postinfection, cells were washed 3 times with ice-cold PBS to remove unbound virus. Cells were lysed with 1 ml TRI-Reagent (Sigma), and genome equivalents were determined via qPCR. All experiments were done in triplicate, and results are presented as mean ± SEM. Bars represent the ratio of MNV genomes to actin compared to mean KO (BV2) or WT (BJAB) results.

Binding of CD300LF to MNV capsid proteins and its role in MNV attachment to host cells have been established (13, 14). To investigate the cell type-dependent functionality of I/LnJ CD300LF, we performed binding assays to determine how different CD300LF expression affects the attachment of MNV to cells, as described previously (30). BV2 and BJAB cells were chosen for this assay, as they produced the highest viral titer in infection experiments yet showed varied ability to support replication when expressing IL/CD300LF (Fig. 7). MNV bound to BV2-KO cells expressing B6/CD300LF significantly more than to the BV2-KO cells; in contrast, MNV binding to BV2-KO cells expressing IL/CD300LF was comparable to its binding to BV2-KO cells (Fig. 8A). These data suggest that B6/CD300LF mediated the binding between MNV and the BV2-KO cells that but IL/CD300LF could not, which is consistent with the nonfunctionality of IL/CD300LF as an MNV receptor in BV2-KO cells (Fig. 4). On the contrary, MNV binding to BJAB cells expressing IL/CD300LF was significantly greater than its binding to WT BJABs, although less than its binding to BJAB cells expressing B6/CD300LF (Fig. 8B). The result was in line with the MNV replication of the BJAB cells expressing CD300LF proteins (Fig. 7D). Collectively, these data suggest that different binding of IL/CD300LF to MNV virions in different cell types might be responsible for the cell type-dependent functionality of I/LnJ CD300LF.

## DISCUSSION

Previous studies have demonstrated that expression of murine CD300LF is necessary and sufficient for MNV infection; indeed, deletion of *Cd300lf* in C57BL/6J mice or in BV2 cells, a microglial cell line derived from C57BL/6 mice, makes them resistant to MNV infection, and expression of CD300LF in HeLa cells confers MNV susceptibility to human cells (9). Here, we show that BMDMs from the I/LnJ mouse strain are resistant to MNV infection while susceptible to a range of other viruses and that this resistance is due to polymorphisms in the extracellular domain of CD300LF in the I/LnJ mouse strain. Published amino acid sequences of CD300LF indicate significant variation among different mouse strains, including in regions important for MNV attachment and entry (9, 11). We identified a series of four amino acids at the CC′ loop which swapped the MNV susceptibility phenotype when exchanged between C57BL/6J and I/LnJ CD300LF. Surprisingly, I/LnJ CD300LF could function as an MNV entry factor when expressed in MEFs and in 293T, HeLa, and BJAB cells, although it could not do so when expressed in BV2 CD300lf-KO or Jurkat T-cell lines. Collectively, our data suggest that cell type-specific factors can modulate utilization of I/LnJ CD300LF in MNV entry.

Public sequence data for various mouse strains indicate that CD300LF proteins broadly segregate into two distinct groups: C57BL/6J-like, such as BALB/cJ and FVB/NJ, and I/LnJ-like, such as C3H/HeJ and CBA/J (aligned in Table 2). Comparatively fewer strains express an intermediate CD300LF like CAST/EiJ. Since the origin and lineage history of inbred mouse strains are not well documented (33), it is difficult to speculate on whether this clustering traces back to an evolutionary pressure in nature or a single divergence point. Evasion of MNV infection might be a selective pressure to drive the divergence of CD300LF proteins. However, as MNV infection in immunocompetent mice is not lethal (1), it is possible that MNV infection would not have exerted a selective pressure strong enough to cause this kind of divergence. Furthermore, considering that there are many cell types known to be infected by MNV *in vivo* (e.g., macrophages, dendritic cells, tuft cells, B cells, and T cells) (25) and that I/LnJ CD300LF could function as an MNV receptor in a cell type-specific manner, it is likely that even the mouse strains containing an I/LnJ-like *Cd300lf* allele are susceptible to MNV infection *in vivo*. Indeed, the C3H/HeJ mouse strain has a CD300LF identical to that of the I/LnJ strain, and it was previously shown to be infected with MNV (34). Our preliminary data also suggest that I/LnJ mice are infectible with MNV, even though I/LnJ BMDMs are not infectible with MNV (Fig. 1 and 2), potentially corresponding with a change in tissue tropism (30, 35). Based on the data shown in Fig. 7, we would predict that I/LnJ B cells are susceptible to MNV infection. Therefore, substantive discussion on the natural variation of CD300LF, including its potential causes and consequences, would require a robust understanding of its function outside the context of infection.

**TABLE 2:**
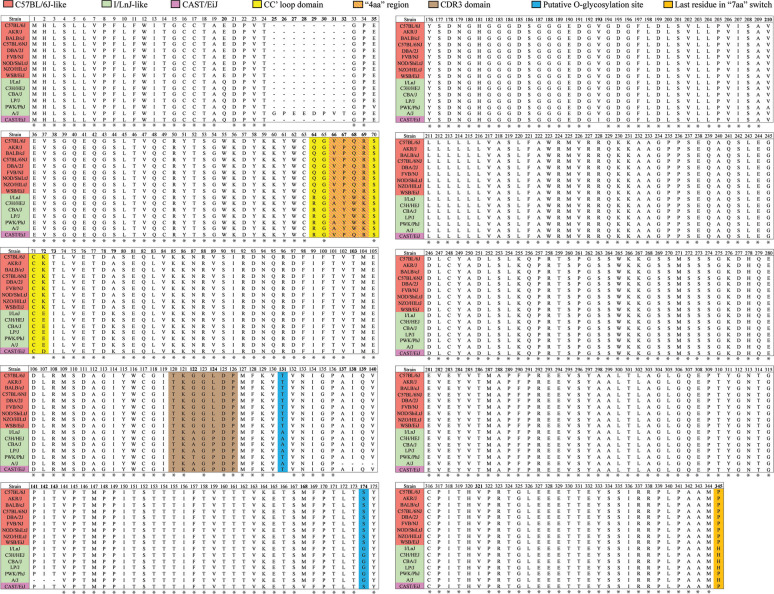
Alignment of CD300LF coding sequences^*a*^

a Available protein sequences for CD300LF in the indicated mouse line, grouped based on their similarity to the sequence of C57BL/6J or I/LnJ CD300LF. Asterisks indicate an amino acid that is common among all sequences.

Although a significant amount of work has been done tracing the evolutionary lineage of the CD300 family, the exact ligands, specific function, and redundancy of function between members is not well understood (36). It is also unclear whether the 4-amino-acid difference in the CC′ loop between mouse strains impacts the cellular functions of CD300LF. It is useful to note that in each of our experiments in which the “4aa” exchange was made (i.e., 7aa and 4aa experiments), the C57BL/6J background-containing allele showed a severe attenuation in ability to confer infectivity, rather than a complete loss of function. This suggests that while the CC′ loop contributes substantially to conferment of MNV susceptibility, it is likely that other sections of the CD300LF protein that differ between the C57BL/6J and I/LnJ alleles also contribute. Further study on the structure and function of CD300LF and CD300 family members in general is warranted.

The most surprising finding in our study is the functionality of I/LnJ CD300LF as an MNV entry factor in a cell type-dependent manner. We consider the following three possibilities for this cell type-specific functionality. First, there may be differential binding of ligands. Other groups have posited that the binding pocket flanked by the CC′ and CDR3 loops may function to augment MNV attachment to CD300LF through the binding of a soluble factor(s), including ceramide (11, 37). Given the proximity of the 4 amino acids to the lipid binding CC′ loop, the polymorphism could impact the ligand binding capacity of CD300LF, such that the CD300LF-localized concentration of that ligand or local cell surface density could differ between cell types and consequently affect functionality for MNV entry. Second, there may be cell type-specific modulators of MNV entry. Positive modifiers of MNV entry would likely be cell type-specific coreceptors. A classic example is HIV entry, in which the virus requires both its receptor, CD4, and a coreceptor in order to successfully enter a host cell (38). These coreceptors, CXCR4 and CCR5, contribute substantially to the host cell tropism of different strains of HIV (39–45). For MNV, the I/LnJ CD300LF might be unable to interact with the macrophage-/T-cell-specific cofactor, while maintaining its interaction with alternative coreceptors expressed in other cell types. Lastly, there may be cell type-specific inhibitors of MNV entry which can interact with I/LnJ CD300LF but not with C57BL/6J CD300LF. A recent study into critical factors for entry of MNV into macrophages and dendritic cells identified several proteins, including CD98 and CD36, that influence attachment and could potentially fulfill such a role (46). All these three possibilities are plausible, and future studies will investigate these possibilities and attempt to identify this modulatory factor(s).

## MATERIALS AND METHODS

### Cloning.

CD300LF cDNAs of mouse strains C57BL/6J and I/LnJ were synthesized based on public sequence databases (http://www.ensembl.org and http://www.informatics.jax.org) and cloned into pCDH-MCS-T2A-copGFP-MSCV (System Biosciences, CD523A-1) and pLenti-CMV-Puro-DEST (w118-1; Addgene plasmid number 17452). To add an HA-epitope at the N terminus of the CD300LF protein after cleavage of the signal peptide, each *Cd300lf* sequence was modified as follows: C57BL/6J, …ACGGCTtacccatacgatgttccagattacgctGAGGAT… (…TAypydvpdyaED…); I/LnJ, …ACGGCTtacccatacgatgttccagattacgctCAGGAT… (…TAypydvpdyaQD…). Lowercase letters indicate the nucleotide and amino acid sequences of inserted HA tag. Specific amino acid swapping mutants (B6/CD300LF-7aa, IL/CD300LF-7aa, B6/CD300LF-4aa, and IL/CD300LF-4aa) were also synthesized and cloned into pLenti-CMV-Puro-DEST (w118-1). The swapping mutants of potential glycosylation sites (B6/CD300LF S174G, B6/CD300LF T131A, IL/CD300LF G174S, and IL/CD300LF A131T) were generated using the QuikChange XL site-directed mutagenesis kit (Agilent) according to the manufacturer’s instructions. The mutated sequences are as follows: C57BL/6J CD300LF-7aa mutant, …CAAGGAGTTCCTCAGAGATCATGT… → …CAAGGAGcTtaTtgGAaATCATGT… (…QGVPQRSC… > …QGaywkSC…), …AAAGTTACTGTGAAC… → …AAAGTTgCTGTGAAC… (…KVTVN… → …KVaVN…), …CTGACTAGCTACTAC… → …CTGACTgGCTACTAC… (…LTSYY… → …LTgYY…), and …GCCATGCCT → …GCCATGcaT (…AMP → …AMh); I/LnJ C300LF-7aa mutant, …CGAGGAGCTTATTGGAAATCATGT… → …CGAGGAGtTccTcaGAgATCATGT… (…RGAYWKSC… → …RGvpqrSC…), …AAAGTTGCTGTGAAC… → …AAAGTTaCTGTGAAC… (…KVAVN… → …KVtVN…), …CTGACCGGCTACTAC… → …CTGACCtcCTACTAC… (…LTGYY… → …LTsYY…), and …GCCATGCAT → …GCCATGCcT (…AMH → …AMp). Lowercase letters indicate the nucleotide and amino acid sequences of the mutants. The sequences of all constructs were checked and confirmed upon cloning via sequencing.

### Cells.

BMDMs were prepared as described previously (47) from mice provided by Tatyana Golovkina at The University of Chicago. Briefly, bone marrow was isolated from femurs and tibias of 6- to 8-week-old mice and plated in non-tissue culture-treated dishes in 10 ml of BMDM medium. Cells were supplemented with fresh medium on day 4 and seeded for experiments on day 7. Wild-type (WT) and *Cd300lf*^−/−^ BV2 cells, mouse embryonic fibroblasts (MEFs), and 293T cells were provided by Herbert “Skip” Virgin (Washington University, St. Louis, MO). HeLa cells were purchased from ATCC. We obtained Jurkat (JRT3.5) cells from Erin Adams (The University of Chicago) and BJAB cells from Stephanie Karst (University of Florida).

### Viruses.

MNV-1.CW3 (herein referred to as CW3) was produced by transfection of 1 × 10^6^ 293T cells with 2.5 μg of a cDNA clone containing the genome of CW3 (48). Cells were then incubated at 37°C for 48 h, frozen at –80°C, thawed, and passed through a 0.45-μm filter. Five hundred microliters of the resulting filtered virus was used to infect 1 × 10^6^ WT BV2 cells. Inoculated cells were incubated until ∼90% cytopathic effect (CPE) was observed. Two 15-cm tissue culture-treated plates were then seeded with 1.5 × 10^7^ BV2 cells, then inoculated with 50 μl of virus, and incubated at 37°C until ∼90% CPE was observed. These cells were frozen at –80°C and thawed. Cell lysates were pooled and centrifuged at 3,000 rpm for 20 min to remove cell debris. The supernatants were ultracentrifuged at 123,918.9 × *g* for 3 h in order to concentrate virus. The resulting pellet was resuspended in Dulbecco modified Eagle medium (DMEM) supplemented to contain 10 mM HEPES (Mediatech, 25-060-CI), 1× MEM nonessential amino acids (Mediatech, 25-025-CI), 100 U/ml each of penicillin and streptomycin (Mediatech, 30-002-CI), and 10% fetal bovine serum (Biowest, US1520) and frozen at –80°C until use. Passage 2 stocks of MNV strains MNV-1.RVSS (RVSS) and MNV-1.CW1 (CW1) and passage 5 stocks of MNV strain GV/CR3/2005/USA (CR3) were used for infection as described for Fig. 2 (30, 31). For each passage, inoculated cells were incubated at 37°C for near-complete cell death and frozen at –80°C. Thawed cell lysates were clarified by centrifugation, and supernatant was used to inoculate new cells for amplification. Encephalomyocarditis virus (EMCV) and murine gammaherpesvirus 68 (MHV-68) were provided by Marco Colonna and Herbert “Skip” Virgin at Washington University (St. Louis, MO), respectively. EMCV and MHV-68 viral stocks were further passaged and amplified in L929 and MEF cells, respectively.

### Viral infection.

All infections were performed as described previously (49). Briefly, 1 × 10^5^ cells were seeded per well in 24-well tissue culture-treated plates. At 24 h after seeding, either the medium was replaced with virus-containing inoculum (adherent cell lines) or a concentrated sample of virus was added to the medium (nonadherent cells) at the indicated MOI. Adherent cells were incubated in virus-containing inoculum for 30 min, washed twice with phosphate-buffered saline (PBS), and replenished with fresh medium. Nonadherent cells were inoculated with 50 μl of concentrated viral stock and mixed by gentle pipetting to achieve the indicated MOI. Cells were then washed and pelleted twice to remove inoculum, and infected cells were harvested at the indicated time points by freezing at –80°C for median tissue culture infectious dose (TCID_50_) analysis.

### TCID_50_ assay.

TCID_50_ assays were performed as reported previously (50). Briefly, inoculated cells were frozen at –80°C to lyse the cells via a cycle of freeze and thaw. Lysates were serially diluted 10-fold in cell growth medium. The samples were then added to BV2 cells seeded in a 96-well plate. Eight wells of cells were inoculated per dilution and incubated for 5 days. The TCID_50_ was calculated by determining the dilution required to show cytopathic effect in 4 out of 8 wells, according to a standard protocol (50). Briefly, the following formula was used to calculate proportionate distance (−PD): [(% positive at or above 50%) – 50%]/[(% positive at or above 50%) – (% positive below 50%)]. The PD was then used to calculate log TCID_50_ using the formula (log dilution at or above 50%) + (−PD). This log value was then used to express the virus titer as TCID_50_/unit volume, e.g., TCID_50_/ml.

### Western blotting.

Western blotting was performed as described previously (47). In short, total cell lysates were harvested in protein sample buffer (0.1 M Tris [pH 6.8], 4% SDS, 4 mM EDTA, 286 mM 2-mercaptoethanol, 3.2 M glycerol, 0.05% bromophenol blue), and proteins were resolved by SDS-PAGE. Proteins were then transferred onto polyvinylidene difluoride (PVDF) membranes, probed with primary antibody diluted in PBS–0.1% Tween 20 (PBST) containing 5% skim milk overnight at 4°C, and then stained with secondary antibody diluted in PBST with 5% skim milk for 1 h at room temperature (RT). Probed proteins were detected using ECL reagents on a ChemiDoc system with Image Lab software (Bio-Rad). The following antibodies were used: mouse anti-HA (Frank W. Fitch Monoclonal Antibody Facility, The University of Chicago, clone 12CA5), goat anti-mouse CD300LF (R&D systems, AF2788), horseradish peroxidase (HRP)-conjugated mouse anti-beta-actin (Santa Cruz Biotechnology, sc-47778), and HRP-conjugated goat anti-mouse (BioLegend, number 405306).

### Flow cytometry.

To examine the cell surface expression of HA-tagged CD300LF mutants, cells (1 × 10^6^ cells/well in 6-well plates) were gently detached by a 5-min incubation with 600 μl of 0.5 mM EDTA and gentle scraping. At this point, cells were centrifuged for 5 min at 1,000 rpm. Cells were then washed in PBS containing 2% fetal bovine serum (FBS) and 1 mM EDTA. Cells were resuspended in wash buffer containing 1% FcR blocker, stained with mouse anti-HA antibody on ice for 30 min, washed 5 times, stained with donkey anti-mouse antibody conjugated with Alexa Fluor 647 for 30 min at RT, and washed again 5 times. Cells were then immediately analyzed with a BD LSRFortessa cell analyzer. Untransduced, unstained samples stained only with primary antibody, samples stained only with secondary antibody, and samples incubated in primary isotype control (mouse IgG2b k isotype; BioLegend) antibodies were used as controls.

### Lentiviral transduction.

A modified version of lentiviral vector pCDH-MCS-T2A-copGFP-MSCV was used to express CD300LF alleles in C57BL/6J and I/LnJ BMDMs, as described previously (47, 49). Lentivirus was generated by transfecting lentiviral vectors with a packaging vector (psPAX2) and a pseudotyping vector (pCMV-MD2.G) into 293T cells via a standard calcium phosphate method. A third-generation lentiviral vector, pLenti-CMV-Puro-DEST (w118-1), was used to express HA-tagged CD300LF alleles in all immortalized cell lines used. Lentivirus was generated by transfecting lentiviral vectors with HIV gag/pol (pMDLg/pRRE), rev (pRSV-rev), and pseudotyping vector (pCMV-MD2.G). Supernatants were collected at 24 and 48 h posttransfection, filtered through a 0.45-μm filter (Millipore), and added to the indicated cells. After 48 h, cells were selected with puromycin (2 μg/ml for HeLa cells and 3 μg/ml for all non-HeLa cells).

### Binding assays.

Binding experiments were performed in accordance with previously published assays (12). Briefly, 1 × 10^5^ cells were seeded in 12-well plates, and 24 h later, the cells were inoculated with MNV (CW3) at an MOI of 2 TCID_50_/cell and incubated at 4°C for 1 h with gentle rocking. Cells were then washed three times with ice-cold PBS to remove unbound virus. BJAB cells were pelleted between washes via centrifugation. Cells were then lysed with 1 ml TRI-Reagent (Sigma), and RNA was extracted according to the manufacturer’s instructions. Genome equivalents were determined via quantitative PCR (qPCR) as described previously (47).

### Statistical Analysis.

All statistical analyses were performed in GraphPad Prism using two-tailed, unpaired *t* tests. All differences not specifically indicated as significant were not significant (*P* > 0.05). Significant values are indicated as follows *, *P* < 0.05; **, *P* < 0.01; ***, *P* < 0.001; ****, *P* < 0.0001.
